# Acquisition of rifabutin resistance by a rifampicin resistant mutant of *Mycobacterium tuberculosis *involves an unusual spectrum of mutations and elevated frequency

**DOI:** 10.1186/1476-0711-4-9

**Published:** 2005-06-15

**Authors:** Richard M Anthony, Anja RJ Schuitema, Indra L Bergval, Tim J Brown, Linda Oskam, Paul R Klatser

**Affiliations:** 1 KIT (Royal Tropical Institute) Biomedical Research, Meibergdreef 39, 1105 AZ Amsterdam, The Netherlands; 2 Health Protection Agency, Mycobacterium Reference Unit, King's College Hospital (Dulwich), London, UK

## Abstract

**Background:**

Mutations in a small region of the *rpoB *gene are responsible for most rifamycin resistance in *Mycobacterium tuberculosis*. In this study we have sequentially generated resistant strains to first rifampicin and then rifabutin. Portions of the *rpoB *gene were sequenced from 131 randomly selected mutants. Second round selection resulted in a changed frequency of specific mutations.

**Methods:**

*Mycobacterium tuberculosis *(strain Mtb72) rifamycin resistant mutants were selected *in vitro *with either rifampicin or rifabutin. One mutant R190 (*rpoB *S522L) selected with rifampicin had a rifampicin MIC of 32 μg/ml but remained sensitive to rifabutin (MIC<0.8 μg/ml). This mutant was subjected to a second round of selection with rifabutin.

**Results:**

All 105 first round resistant mutants derived from the parent strain (Mtb72) screened acquired mutations within the 81 bp *rpoB *hotspot. When the rifampicin resistant but rifabutin sensitive S522L mutant was subjected to a second round of selection, single additional *rpoB *mutations were identified in 24 (92%) of 26 second round mutants studied, but 14 (54%) of these strains contained mutations outside the 81 bp hotspot (codons 144, 146, 148, 505). Additionally, spontaneous rifabutin resistant mutants were produced at >10 times the frequency by the S522L mutant than the parent strain.

**Conclusion:**

First round selection of mutation S522L with rifampicin increased the frequency and changed the spectrum of mutations identified after selection with rifabutin.

## Introduction

It has been estimated that one third of the World's population is infected with *Mycobacterium tuberculosis *(MTB) resulting in 2 million deaths annually. In uncomplicated cases short course therapy (6 months) using a multiple-drug regimen is highly effective. An essential component of this regimen is rifampicin (RIF). In MTB resistance to antimicrobial agents appears to be solely due to spontaneous mutation, as no horizontal transfer of genetic elements carrying a resistance genotype has been described. For this reason MTB strains or sub-populations with an unusual spectrum or rate of mutations are of considerable interest [1-3] as potentially they are more prone to develop resistance to antimicrobial drugs.

Mutations within an 81-bp locus of MTB *rpoB *have been seen in almost all (> = 95%) rifamycin resistant isolates, whether they be clinical [4] or laboratory generated mutants [5]. These mutations are not only markers of resistance as the region of RpoB coded for by this locus has been shown to bind rifamycins and mutations in this region allow RpoB to function in the presence of rifamycins [6]. Although mutations associated with RIF resistance have been reported throughout this locus, mutations in two codons account for approximately 70% of all mutants identified (codon-526 approximately 20%, and codon-531 approximately 50%), from most collections of isolates studied.

Rifabutin (RFB) is a second therapeutically useful rifamycin, which is often used when *M. avium / intracellulare *(MAI) infection is suspected or confirmed. RIF and RFB resistances are not invariably cross-resistant although the most common mutations (codons 526 and 531) are reported to result in high-level resistance to both drugs. Certain mutations in other codons, notably 511, 516, and specific mutations in 522, however, have been reported to result in lower level resistance to RIF only [7,8].

Here we report the generation and characterisation (sequencing subgenic fragments of the *rpoB *gene) of two sets of rifamycin resistant mutants selected from a single parent strain using either RIF or RFB. Mutations that confer resistance to RIF but not RFB cannot be selected for by RFB. Thus certain mutations are not possible when RFB is used for the selection of mutant cells and different selections and/or frequencies of mutations would be expected. Strains with mutations conferring resistance to RIF only can be subjected to a second round of selection with RFB and the type and frequency of resistant mutants compared to that of the sensitive parent strain [2]. In this study the single isolate identified with resistance to RIF only, was subjected to a second round of selection with RFB; after which *rpoB *gene fragments of 26 randomly selected colonies were re-sequenced in order to identify any additional mutations.

## Methods

### Strain used

*Mycobacterium tuberculosis *Mtb72 (ATCC35801) belongs to the Haarlem genotype and was the parent strain of all isolates generated in this study.

### Generation and selection of mutants

Bacteria were cultured in Middlebrook 7H9 (Difco) broth for 14 days in a shaking incubator at 37°C. Then 0.5 ml of broth was plated out onto Middlebrook 7H11 (Difco) media containing either RIF 8 μg/ml (Sigma) or RFB 0.8 μg/ml (Pharmacia Corporation, MI, USA) for the first round selection or 0.8 μg/ml and 8 μg/ml RFB for the second round selection. These plates were incubated at 37°C and colonies picked between 21 and 42 days culture. Selected colonies were streaked on drug containing media to confirm the resistant phenotype (RFB 0.8 μg/ml, RIF 8 μg/ml).

**MICs **were measured on Middlebrook 7H11 plates containing serial dilutions RIF (512 μg/ml to 4 μg/ml) or RFB (128 μg/ml to 1 μg/ml) for 24 isolates chosen so as to represent the full range of genotypes identified. Only strains showing growth of more than 1% of the inoculate were scored as resistant.

### PCR/ DNA sequencing

A 271 bp fragment of the *rpoB *gene containing the 81 bp mutation hotspot (Cluster I) was amplified and sequenced from the parent and all 131 mutants studied. Crude DNA extracts were prepared for PCR by heating cell suspensions to 95°C for 20 min in TE buffer containing 1% Triton X-100. PCR of *rpoB *cluster I was carried out on all 132 isolates included in the study, in 20 μl volumes, containing 2 μl 10× PCR buffer (Bioline Ltd., London, UK); 0.5 unit Taq-DNA polymerase (Bioline); 0.5 μl 2 mM dNTP mixture (Bioline); 0.5 μl 20 μM primer mix (containing rpoBP1, 5'ggtcggcatgtcgcggatgg and rpoB1420R, 5'gtagtgcgacgggtgcacgtc) 15.5 μl water and 1 μl of DNA extract. Thermal cycling was performed in a Thermocycler using the following programme 5 min at 95°C, 30 × (30 sec at 95°C, 30 sec at 65°C, 60 sec at 72°C), 5 min at 72°C. The presence of PCR products was confirmed by agarose gel electrophoresis. These products were diluted 1/100 in purified water and sequenced using CEQ Quick Start sequencing kits and a CEQ 8000 instrument (Beckman Coulter, High Wycombe, UK) according to the manufacturer's instructions. The PCR products were sequenced in both directions using the amplification primers given above. All codon numbers are reported using the *E. coli *numbering system.

An additional 365 bp region of the *rpoB *gene (containing codon 176 [10]) from all 26 second round mutants and the two parent strains was also amplified and sequenced using primers rpob7F (cttctccgggtcgatgtcgttg) and rpob7R (CGCGCTTGTCGACGTCAAACTC). PCR was carried out in 25 μl volumes, containing 2.5 μl 10× HotGoldstar PCR buffer (Eurogentec); 2 μl 25 mM MgCl2; 0.15 μl (5 units/μl) HotGoldstar DNA polymerase (Eurogentec); 0.2 μl 25 mM dNTP mixture (Amersham Bioscience); 0.25 μl 10 μM of each primer, 18.6 μl water and 1 μl of crude DNA extract. Thermal cycling was performed in a Thermocycler using the following programme: 10 min at 95°C, 35× (30 sec at 96°C, 30 sec at 60°C, 60 sec at 72°C). The presence of PCR products was confirmed by agarose gel electrophoresis. These products were diluted 1/10 in purified water and sequenced using the dideoxy chain termination method with the Big Dye Terminator cycle sequencing Kit (Applied Biosystems, CA, USA). PCR was carried out, using quarter reactions with either the forward or reverse primer. Sequence analysis was performed on a 310 Genetic Analyzer (Applied Biosystems).

**Mutation frequency **was measured by plating 1.5 ml of two entirely independent exponentially growing bacterial cultures in antibiotic free Middlebrook 7H9 broth onto a series of Middlebrook 7H11 plates, one set containing 0.8 μg/ml and one set containing 8 μg/ml RFB. Decimal dilution series of each of these cultures were prepared and plated onto non-selective Middlebrook 7H11 plates, the CFU present on these plates was used to calculate the inoculum size. The mutation frequency, calculated separately for each drug concentration used, was the number of resistant colonies / number of CFU inoculated.

## Results

Mutations in the 81-bp locus of the MTB *rpoB *gene were detected in cluster I (between codons 512 and 532) in all 105 first round resistant mutants that were sequenced (Table 1); no mutations were detected in the parent strain and two blinded wild type isolates included in this panel as sequencing controls.

**Table 1:** Distribution of rpoB spontaneous mutations identified after *in vitro *exposure to rifampicin (8 μg/ml) or rifabutin (0.8 μg/ml).

	**Number of mutants (%)**									
**Selective agent (Strain)**	CAA> CTA Q513L	TCG> TTG S522L	CAC> GAC H526D	CAC> TAC H526Y	CAC> CGC H526R	CAC> CCC H526P	TCG> TTG S531L	TCG> TGG S531W	**Others**	**No. tested**

Rifabutin 0.8 μg/ml	1	0	5	27	6	0	6	1	1*	
(Mtb72)	(2)	(0)	(11)	(60)	(13)	(0)	(13)	(2)	(2)	**47**
Rifampicin 8 μg/ml	0	1	6	26	4	1	20	0	0	
(Mtb72)	(0)	(2)	(11)	(45)	(7)	(2)	(35)	(0)	(0)	**58**

* = 525 ACC>ACG + 526 CAC>CCC + 527 AAG>CAG

Two regions of the *rpoB *gene from 26 randomly selected RFB second round mutants were sequenced (Table 2). Single mutations in addition to the original S522L mutation in the *rpoB *gene were identified in 24 of the 26 strains (92%). Twelve strains had mutations between codons 505 and 531, and 12 strains had mutations between codons 144 and 148, the final two strains did not have any additional mutations in the regions sequenced. No (additional) mutations were detected in the two parent strains (Mtb72 and R190) sequenced as controls.

**Table 2:** Additional mutations acquired in 26 randomly selected colonies of rifampicin resistant isolate R190 (S522L) after a second round of selection with rifabutin.

Strain	Conc. of RFB used for selection μg/ml	Additional mutations identified* codons 505–531 (Cluster I 507–533)	Additional mutations identified* codons 144–148
1	0.8	F505L TTC>TTA	None
2	0.8	F505L TTC>TTG	None
3	0.8	S512G AGC>GGC	None
4	8	S512G AGC>GGC	None
5	8	S512G AGC>GGC	None
6	8	S512G AGC>GGC	None
7	0.8	H526Y CAC>TAC	None
8	8	H526Y CAC>TAC	None
9	0.8	S531L TCG>TTG	None
10	0.8	S531L TCG>TTG	None
11	0.8	S531L TCG>TTG	None
12	0.8	S531L TCG>TTG	None
13	8	None	V144M GTG>ATG
14	8	None	V144M GTG>ATG
15	8	None	V144G GTG>GGG
16	0.8	None	V146F GTC>TTC
17	0.8	None	V146F GTC>TTC
18	0.8	None	V146F GTC>TTC
19	8	None	V146F GTC>TTC
20	8	None	V146F GTC>TTC
21	8	None	V146F GTC>TTC
22	8	None	Q148H CAG>CAC
23	0.8	None	Q148H CAG>CAC
24	0.8	None	Q148H CAG>CAC
25	0.8	None	None
26	0.8	None	None

*All isolates retained the original S522L TCG>TTG mutation from their R190 parent and had at most a single additional mutation identified. Codons are numbered according to the *E. coli *numbering system.

The MICs of selected isolates representing the spectrum of mutations obtained are shown in table 3. The single strain identified as resistant to rifampicin (MIC 32 μg/ml) but sensitive to rifabutin (strain R190, MIC<0.8 μg/ml, mutation S522L) was subjected to a second round of selection using RFB, in parallel with the parent strain (Mtb72). The frequency of mutants produced after 28 days incubation at 37°C by R190 (S522L) on two different RFB concentrations was compared to the mutation frequency of the parent strain. After selection with 0.8 μg/ml RFB the parent strain generated RFB resistant mutants with a frequency of 1.25 × 10^-7 ^colonies per CFU plated and the R190 (S522L) mutant generated RFB resistant mutants with a frequency of 1.93 × 10^-6 ^per CFU plated. After selection with 8.0 μg/ml RFB the parent strain generated RFB resistant mutants with a frequency of 1.42 × 10^-8 ^colonies per CFU plated and the R190 mutant generated RFB resistant mutants with a frequency of 1.7 × 10^-7 ^per CFU plated. Thus, spontaneous RFB mutants were generated by R190 with 11.6 times the frequency of the parent strain with 8 μg/ml RFB and 15.4 times the frequency of the parent strain with 0.8 μg/ml RFB.

**Table 3:** Rifampicin and rifabutin MICs of selected first generation *in vitro *mutants.

***rpoB *Mutation**	**Number of Isolates**	**RIF MIC μg/ml**	**Number of Isolates**	**RFB MIC μg/ml**
Wild	2	<4	2	<0.8
Q513L	1	256	1	>128
S522L	1	32	1	<0.8
H526Y	3	256	1	>128
	1	128	2	128
			1	64
H526D	3	256	1	>128
	1	128	3	128
H526R	1	512	5	128
	3	256		
	1	128		
H526P	1	256	1	>128
S531L	4	256	1	>128
			2	128
			1	64
S531W	1	256	1	128

## Discussion

In this study a single *M. tuberculosis *isolate (R190), with a S522L mutation, was detected that was resistant to RIF (MIC 32 μg/ml) but remained sensitive to RFB (MIC <0.8 μg/ml) (Table 3). Specific mutations in codon 522 have previously been shown to result in only low level RFB resistance from clinical isolates of MTB [7,9]. The distribution of second round RFB mutations from R190 was strikingly different from that obtained from the parent strain (Table 2) and the frequency of resistant mutants increased > 10 fold when measured on two separate occasions. Additional second round mutations in the *rpoB *gene were identified in 24 of 26 (92%) of the R190 second round RFB resistant mutants (Table 2), only six of which (23%) were similar to those seen in the first round selection (either in codon 526 or 531). A single mutant with a change in codon 512 at the beginning of cluster I was also identified. Mutations in codon 512 have only been reported previously in association with additional mutations [10] as in this study (S512G + S522L). The remaining mutations were outside cluster 1, codons 144, 146, 148, and just before cluster I in codon 505 (Table 2).

This dramatic change in mutations identified indicates either, the range of viable SNPs resulting in high-level RFB resistance was significantly altered by the presence of the S522L mutation, or the range of spontaneous mutations occurring in this strain changed. Thus, it is possible, for example, that the mutations in codon 505 occurred in both cases but alone would not have resulted in resistance or in the absence of the S522L mutation may be lethal. Interestingly, these results could also be explained by a change in both the frequency and spectrum of spontaneous mutations after the first round of selection, ie. the rate of spontaneous mutations in codons 505, 512, 144, 146, and 148, has increased after the first round of selection. As 9 different codon changes were identified among the 26 isolates tested (Table 2), from two independent experiments (drug concentrations), a single spontaneous mutant in an early generation of this culture (a "Jackpot" mutation) [11] cannot explain this result.

The distribution of *in vitro *first round spontaneous mutations in *M. tuberculosis *has previously been reported [5] and 7 of the 9 codon changes identified were also seen in this study. The most striking difference between this data and the first round selection data presented here is the higher frequency of the C>T H526Y mutation in our study with both RIF and RFB. Some variation between random selections of mutants would be expected and methodological differences, notably the use of a different bacterial strain [5,12], probably contributed to this effect.

Mutations in codons 526 and 531 predominate in most of the published studies but there is also some indication that certain strains may be prone to develop specific mutations [4,12] and marked differences in the distribution of mutations have been observed in different geographical locations [4,13]. Our data from the first round selection (Table 1) suggest that for the strain and conditions we used RFB may be more likely to select for C>T H526Y mutations than rifampicin.

All first round mutations identified in this study have been reported previously from clinical isolates [4,5,8-10,14] except the one triple mutant identified (table 1). The second round mutations seen in codons 505, 512, 144, and 148 present in addition to the S522L mutation (Table 2), have to our knowledge not been reported previously from *in vitro *or clinical isolates. Although, it should be noted that these mutations lie outside the 81 bp hotspot region in a region of the *rpoB *gene that has been subjected to much less extensive investigation.

The possibility of an MTB strain with altered or raised mutation rate is important. Selection of a mutator phenotype is recognised as a consequence of antibiotic challenge in many bacterial species [11,15]. The selection of strains with increased mutation rates will result in a greater chance of acquiring resistance to other drugs but may also impact on the pathogenicity of the strain. It has been reported that many MDR-MTB strains have, at least initially, reduced pathogenicity [16] and mutations in three putative mutator genes as well as evidence for a reversion back to a more pathogenic non-mutator state after the acquisition of drug resistance has been reported in W-Beijing strains [3]. However, some *rpoB *mutations are associated with only a modest decrease in *in vitro *fitness [17]. Interestingly, induction of the proposed MTB error prone DNA repair enzyme was associated with survival of the bacteria *in vivo *[1], so the effect of a mutator phenotype on pathogenicity is difficult to predict [18].

In conclusion, the presence of a different spectrum of secondary rifabutin mutations implies that either, the mutation rate of individual mutations has changed, due to a defect in DNA repair or replication, or that additional spontaneous mutations are viable (and lead to resistance) in the presence of the S522L mutation [2]. A further consequence of this observation is that a proportion clinical isolates with mutations in cluster 1 of the *rpoB *gene associated with rifampicin resistance only may in fact be resistant to rifabutin as a consequence of addition mutations in other regions of this gene. We believe further study is warranted, of this and similar strains which should include generating mutants to other antimicrobials and measuring mutation rates [19,20], allowing the contribution of each of these possible explanations to be explored. The details of how resistance mutations arise would be valuable when formulating standard treatment regimens with the aim of minimising the emergence of resistance in treated populations [21].

## References

[B1] Boshoff HIM, Reed MB, Barry III CE, Mizrahi V (2003). DnaE2 polymerase contributes to In Vivo survival and the emergence of drug resistance in *Mycobacterium tuberculosis*. Cell.

[B2] Mokrousov I (2004). Multiple *rpoB *mutants of *Mycobacterium tuberculosis *and second-order selection. Emerg Infect Dis.

[B3] Rad ME, Bifani P, Martin C, Kremer K, Samper S, Rauzier J, Kreiswirth B, Blazquez J, Jouan M, van Soolingen D, Gicquel B (2003). Mutations in putative mutator genes of *Mycobacterium tuberculosis *strains of the W-Beijing family. Emerg Infect Dis.

[B4] Qian L, Abe C, Ping-Tao L, Sang-Nae C, Wang S, Douglas JT (2002). *rpoB *Genotypes of *Mycobacterium tuberculosis *Beijing family isolates from East Asian countries. J Clin Microbiol.

[B5] Morlock GP, Plikaytis BB, Crawford JT (2000). Characterization of spontaneous, *in vitro *selected, rifampin-resistant mutants of *Mycobacterium tuberculosis *strain H37Rv. Antimicrob Agents Chemother.

[B6] Campbell EA, Korzheva N, Mustaev A (2001). Structural mechanism for rifampicin inhibition of bacterial RNA polymerase. Cell.

[B7] Cavusoglu C, Karaca-Derici Y, Bilgic A (2004). In-vitro activity of rifabutin against rifampicin-resistant *Mycobacterium tuberculosis *isolates with known *rpoB *mutations. Clinical Microbiology and Infection.

[B8] Williams DL, Waguespack C, Eisenach K (1994). Characterization of rifampin resistance in pathogenic mycobacteria. Antimicrob Agents Chemother.

[B9] Yuen LKW, David L, Coloe PJ (1999). Bacteriological and molecular analysis of rifampin-resistant *Mycobacterium tuberculosis *strains in Australia. J Clin Microbiol.

[B10] Heep M, Brandstatter B, Reiger U, Lehn N, Richter E, Rusch-Gerdes S, Niemann S (2001). Frequency of *rpoB *mutations inside and outside the cluster I region in rifampin-resistant clinical *Mycobcterium tuberculosis *isolates. J Clin Microbiol.

[B11] Martinez JL, Baquero F (2000). Mutation frequencies and antibiotic resistance. Antimicrob Agents Chemother.

[B12] Hillemann D, Kubica T, Rusch-Gerdes S, Niemann S (2005). Disequilibrium in distribution of resistance mutations among *Mycobacterial tuberculosis *Beijing and non-beijing strains isolated from patients in Germany. Antimicrob Agents Chemother.

[B13] Mokrousov I, Bhanu NV, Suffys PN, Kadival GV, Yap S-F, Cho S-N, Jordaan AM, Narvskaya O, Singh UB, Gomes HM, Lee H, Kulkarni SP, Lim KC, Khan BK, van Soolingen D, Victor TC, Schouls LM (2004). Multicenter evaluation of reverse line blot assay for detection of drug resistance in *Mycobacterium tuberculosis *clinical isolates. J Microbiol Methods.

[B14] Herrera L, Jimenez S, Valverde A, Garcia-Aranda MA, Juan A, saez-Nieto (2003). Molecular analysis of rifampicin-resistant *Mycobacterium tuberculosis *isolated in Spain (1996–2001). Description of new mutations in the *rpoB *gene and review of the literature. Int J Antimicrob Agents.

[B15] Matic I, Radman M, Tadde FI (1997). Highly variable mutation rates in commensal and pathogenic *Escherichia coli*. Science.

[B16] Gillespie SH (2002). Evolution of drug resistance in *Mycobacterium tuberculosis: *Clinical and molecular perspective. Antimicrob Agents Chemother.

[B17] Billington OJ, McHugh TD, Gillespie SH (1999). Physiological cost of rifampin resistance induced *in vitro *in Mycobacterium. Antimicrob Agents Chemother.

[B18] Tanaka MM, Bergstrom CT, Levin BR (2003). The evolution of mutator genes in bacterial populations: the roles of environmental change and timing. Genetics.

[B19] Luria SE, Delbrück M (1943). Mutations of bacteria from virus sensitivity to virus resistance. Genetics.

[B20] Werngren J, Hoffner SE (2003). Drug-susceptible *Mycobacterium tuberculosis *Beijing genotype does not develop mutation-conferred resistance to rifampin at an elevated rate. J Clin Microbiol.

[B21] Gumbo T, Louie A, Deziel MR, Parsons LM, Salfinger M, Drusano GL (2004). Selection of a moxifloxacin dose that suppresses drug resistance in *Mycobacterium tuberculosis*, by use of an *in vitro *pharmacodynamic infection model and mathematical modeling. J Infect Dis.

